# Accelerating cutaneous healing in a rodent model of type II diabetes utilizing non-invasive focused ultrasound targeted at the spleen

**DOI:** 10.3389/fnins.2022.1039960

**Published:** 2022-11-17

**Authors:** Christine Morton, Victoria Cotero, Jeffrey Ashe, Fiona Ginty, Christopher Puleo

**Affiliations:** ^1^GE Research, Niskayuna, NY, United States; ^2^Biology and Applied Physics, GE Research, Niskayuna, NY, United States

**Keywords:** bioelectronic medicine, nerve stimulation, neuromodulation, therapy, ultrasound, wound care, excisional wounds

## Abstract

Healing of wounds is delayed in Type 2 Diabetes Mellitus (T2DM), and new treatment approaches are urgently needed. Our earlier work showed that splenic pulsed focused ultrasound (pFUS) alters inflammatory cytokines in models of acute endotoxemia and pneumonia *via* modulation of the cholinergic anti-inflammatory pathway (CAP) (ref below). Based on these earlier results, we hypothesized that daily splenic exposure to pFUS during wound healing would accelerate closure rate *via* altered systemic cytokine titers. In this study, we applied non-invasive ultrasound directed to the spleen of a rodent model [Zucker Diabetic Sprague Dawley (ZDSD) rats] of T2DM with full thickness cutaneous excisional wounds in an attempt to accelerate wound healing *via* normalization of T2DM-driven aberrant cytokine expression. Daily (1x/day, Monday-Friday) pFUS pulses were targeted externally to the spleen area for 3 min over the course of 15 days. Wound diameter was measured daily, and levels of cytokines were evaluated in spleen and wound bed lysates. Non-invasive splenic pFUS accelerated wound closure by up to 4.5 days vs. sham controls. The time to heal in all treated groups was comparable to that of healthy rats from previously published studies (ref below), suggesting that the pFUS treatment restored a normal wound healing phenotype to the ZDSD rats. IL-6 was lower in stimulated spleen (-2.24 ± 0.81 Log2FC, *p* = 0.02) while L-selectin was higher in the wound bed of stimulated rodents (2.53 ± 0.72 Log2FC, *p* = 0.003). In summary, splenic pFUS accelerates healing in a T2DM rat model, demonstrating the potential of the method to provide a novel, non-invasive approach for wound care in diabetes.

## Introduction

The cost of care for non-healing wounds in Medicare recipients is over 28 billion as of 2018, with the number of individuals affected being above 8 million and increasing as the population ages (Nussbaum et al., 2018). There is also significant mortality associated with non-healing wounds, with 5-year mortality rates being higher than many common cancers such as prostate and breast (Armstrong et al., 2007). The 5-year mortality rate approaches 50% when amputation is necessary due to necrosis (Hoffmann et al., 2015), which has been attributed to a wound infection rate of around 50% (Mavrogenis et al., 2018). Once a first amputation is necessary, multiple amputations are often required and the 5-year mortality rate rises to over 70% (Hingorani et al., 2016). Chronic wounds, defined as wounds that do not heal within 3 months, tend to have different microflora than healing wounds and are more susceptible to infection, although infection is not a prerequisite to impaired healing (Armstrong et al., 2007; Hingorani et al., 2016; Mavrogenis et al., 2018). Chronic wounds generally fall into the three categories of diabetic foot ulcers (DFU), pressure ulcers (bed sores), venous leg ulcers, and ischemic leg ulcers caused by peripheral arterial disease (PAD) or post thrombotic syndrome (PTS) (Knighton et al., 1986; Mezera and Bureš, 2018). Chronic inflammation, a frequent comorbidity of T2DM, can negatively influence healing by increasing the systemic levels of pro-inflammatory cytokines. DFU represents the most common complication in patients with poor disease control (e.g., accelerated metabolic syndrome, chronic inflammation) affecting more than 25% of those with T2DM (Mavrogenis et al., 2018). Lack of mobility and/or flexibility are often cited by patients with DFUs as reasons for low compliance with foot self-care protocols. Many patients with diabetes report incidences of slow healing or chronic wounds, including DFU, which results in an annual burden on the healthcare system exceeding 20 billion dollars (Harding et al., 2002).

Normal wound healing consists of four phases –hemostasis, inflammation, proliferation, remodeling—and is similar for acute and chronic wounds, albeit with different timing; failure at any one of these stages can lead to non-healing wound (Velnar et al., 2009; Placek et al., 2019). The healing cascade has been extensively reviewed (Velnar et al., 2009; Lindley et al., 2016; Rittié, 2016; Agyare et al., 2018) as well as the causes for chronic non-healing wounds (Menke et al., 2007; Berlanga-Acosta et al., 2014; Han and Ceilley, 2017; Mezera and Bureš, 2018). Briefly, hemostasis, causes the wound site to close by clotting. The inflammatory phase starts with infiltration of the wound site by neutrophils followed by macrophage and the main function is to prevent infection by phagocytosing bacteria and other pathogens, foreign particles, damaged cells. Lymphocytes (T-cells) enter the wound site in the late inflammatory phase. Classical macrophages (M1 phenotype) secrete proinflammatory cytokines which help recruit lymphocytes to the wound bed (Delavary et al., 2011; Placek et al., 2019). Non-classical macrophages (M2 phenotype) secrete anti-inflammatory cytokines which trigger the transition into the proliferation phase (Olingy et al., 2017). The proliferative phase starts on the third day after wounding and lasts for about 2 weeks. Finally, the remodeling/maturation phase is responsible for development of new epithelium and scar tissue formation and can last 1–2 years. This phase also draws the wound together, much like the contraction of muscle cells, and forms a repaired ECM (Xue and Jackson, 2015). Collagen is remodeled and the wound fully closes. The skin is generally at about 80% integrity until this phase is finished and is significantly weaker and prone to reinjury during this time (Witte and Barbul, 1997). Once a scar is formed, the skin never regains the full integrity of uninjured skin and also lacks elasticity leading to hindered movement (Marshall et al., 2018). Any one of these stages can fail, although healing tends to become arrested in the inflammatory stage (Guo and DiPietro, 2010), never progressing to epithelialization and reconstruction.

Advancement in chronic wound treatment (including DFU) has been lacking for decades. The necessity to develop new technology and advance the treatment of chronic DFU wounds is paramount. The last substantial innovation was over 20 years ago in the form of negative pressure wound therapy (NPWT) (Armstrong et al., 2004a; Blume et al., 2008; Kim et al., 2015). Current treatment consists of wound management using techniques such as using moist wound healing (MWH), debridement, growth factor-soaked dressings, and walker casts to off-load the injured regions (Armstrong and Lavery, 1998; Armstrong et al., 2004b; Breuing et al., 2005; Wu et al., 2007; Blume et al., 2008; Biswas et al., 2010; Armstrong and Andros, 2012; Alkahtani et al., 2017). All of these mitigations are aimed at nursing the wound but not treating a root cause such as chronic inflammation (Rosique et al., 2015; Serra et al., 2017). NPWT, which is more efficacious for acute wounds, is still utilized for chronic wounds with limited success (Wukich et al., 2013). Targeted drug therapies are largely non-existent, and antibiotic administration, both topically and systematically, remains the most widely used pharmaceutical approach.

Bioelectronic medicine is a rapidly evolving field with a recent history of around 20 years (Ekmekçi and Kaptan, 2017; Sahyouni et al., 2017, 2019). Historically, electrical stimulation was applied to nerve fibers to elicit a response and in many cases an electrode is surgically implanted to deliver energy to the vagus nerve, which then has a downstream effect (Ekmekçi and Kaptan, 2017). Borovikova et al. (2000) discovered that vagus nerve stimulation could modulate inflammation *via* CAP signaling. CAP, reviewed here (Czura et al., 2007), is a neural pathway that inhibits TNFα production (as well as other pro-inflammatory cytokines) when activated in the spleen, liver and heart. This pathway requires both the vagus nerve and 7α receptors expressed on macrophages, and the anti-inflammatory response is reversed if either are disrupted. Building on these findings and with a desire for less invasive intervention that could be applied in a point of care setting (i.e., non-surgical), our group has developed methods to activate the CAP non-invasively using focused ultrasound targeted specifically to an organ (i.e., spleen) (Cotero et al., 2019). We found that a single ultrasound treatment decreased systemic TNFα in an *in vivo* LPS induced model of endotoxemia within 1 h (Cotero et al., 2019). Additionally, peripheral blood, collected post pFUS stimulation and exposed to LPS *ex vivo*, produced similar results (Cotero et al., 2019). We have also observed systemic effects at sites distant from the stimulated site such as attenuation of inflammation caused by a challenge with Streptococcus Pneumonia (Akhtar et al., 2021).

**FIGURE 1:**
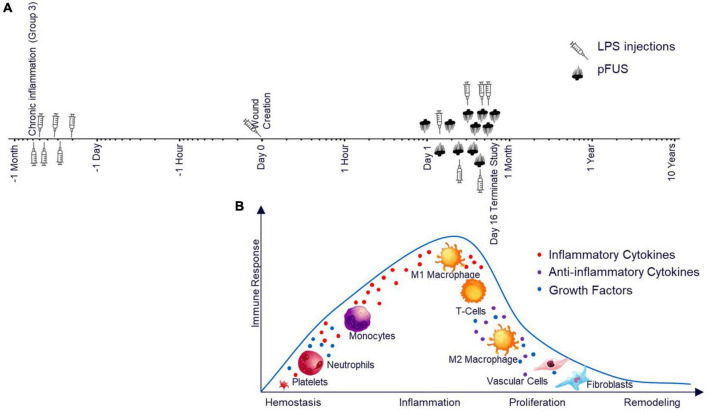
Study timeline and the relationship to the wound healing timeline. **(A)** Study timeline including LPS injections for Group 3 and pFUS stimulation for all groups on a logarithmic scale. **(B)** Expected stages of wound healing after pFUS intervention and the associated immune response time matched to the timeline above. The cell type illustrations in Figure 1B were purchased from iStock.

**FIGURE 2:**
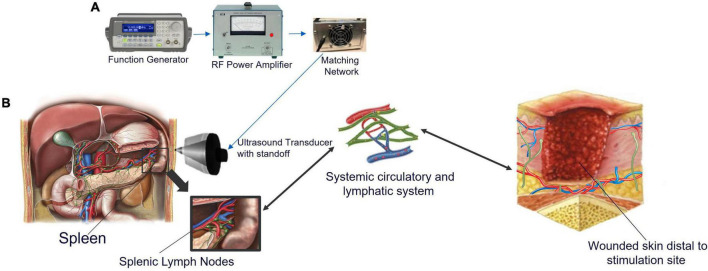
Ultrasound setup and the systemic interaction between the spleen, lymphatic and circulatory systems, and injured skin. **(A)** Images of the components show the function generator, which produces a pulsed sinusoidal waveform. This pulsed sinusoidal waveform is amplified by the RF power amplifier and sent to the impedance-matching network connected to the transducer. The pulse center frequency was 1.1 MHz, the pulse repetition period was 0.5 ms (corresponding to a pulse repetition frequency of 2,000 Hz); the pulse amplitude was 300 mVpp. **(B)** Abdomen showing the location of the spleen and delivery location of pFUS. When non-invasive ultrasound energy is applied to the spleen, cytokine production is modified. Splenic lymph nodes (green), and splenic arteries (red) and veins (blue) are shown in the expansion. Splenic lymph nodes connect to the lymphatic vasculature, which connects the entire lymphatic system, including lymphatics in the skin. Skin resident macrophage release chemoattractant to recruit systemic immune cells through extravasation. When skin is wounded, vessels surrounding the area deliver platelets to achieve homeostasis followed by neutrophils within 1 h to initiate inflammation and start the healing process. Monocytes arrive next and differentiate into M1 macrophage. Efferent lymph vessels help to drain the area of protein rich inflammatory fluid from the interstitium while afferent vessels help supply the area with cytokines and lymphatic leukocytes to aid in healing. The illustrations in Figure 2B were purchased from iStock and Science Source.

In this current study, we assessed the ability of pFUS to accelerate cutaneous wound healing (Figure 1A) *via* stimulation of nerve pathways within the spleen which is based on previous work in which CAP was activated using an electrical implant (Borovikova et al., 2000). Figure 1B aligns the healthy progression of wound healing to the experimental timeline in Figure 1A. Figure 2 depicts the ultrasound set up (Figure 2A) as well as the stimulation site including the relationship between the spleen and skin through both blood and lymph circulation (Figure 2B). We hypothesized that splenic pFUS would alter migration of neutrophils and monocytes/macrophages to the wound bed *via* early inflammation attenuation through cholinergic anti-inflammatory pathway (CAP) modulation (Huston et al., 2009; Swirski et al., 2009), in turn modifying the wound so that it is more amendable to healing. We propose that this is through systemic immune response mediation and altered concentrations of systemic circulating pro-inflammatory molecules (such as TNFα, IL-6).

In this report, we tested Zucker Diabetic Sprague Dawley (ZDSD) rats as a model of delayed healing (Peterson et al., 2015, 2017; Suckow et al., 2017; Sun et al., 2018; Rai et al., 2022). This model has been cited as a preferred model due to the overlapping disease features exhibited in both the ZDSD rats and humans (Reinwald et al., 2009; Rai et al., 2022). We stimulated the spleen of ZDSD rats with full thickness excisional skin wounds up to once a day for 15 days. Our test groups included rats from three age classifications that correspond to different disease progressions of T2DM, from prediabetes to advanced diabetes with comorbidities (Choy et al., 2016). To further model chronic inflammation, a comorbidity of T2DM, the oldest group received an ultralow dose of LPS (5 ng/kg) 3 times a week starting 14 days prior to wound creation (Yuan et al., 2016). An pFUS-induced acceleration in wound closure was observed for all age groups compared to sham controls. While ultrasound has been used in other aspects of chronic wound care such as revascularization (Breuing et al., 2005; Alkahtani et al., 2017; Wiegand et al., 2017), we believe that this is the first demonstration in which ultrasound has been used to stimulate splenic CAP and modulate the immune system distal from the wound site to accelerate healing.

## Materials and methods

### Focused ultrasound setup

The ultrasound system (Figure 2A) used in this study has been described and characterized in detail (Cotero et al., 2019). Briefly, the system consists of a 1.1 MHz, High Intensity Focused Ultrasound (HIFU) transducer and matching network (Sonic Concepts, H106), an RF power amplifier (ENI 350L) and a function generator (Agilent 33120A). The 70-mm diameter HIFU transducer has a spherical face with a 65-mm radius of curvature. The transducer depth of focus is 65 mm. The numerically simulated pressure profile has a full width at half amplitude of 1.8 mm laterally and 12 mm in the depth direction. The HIFU transducer is acoustically coupled to the rat through a 6-cm-tall plastic cone filled with degassed water (Cotero et al., 2019), and acoustical coupling gel.

### Animal studies

All animal studies were approved by the GE Research Institutional Animal Care and Use Committee. Thirty 16–25 week old ZDSD rats were randomized into the following groups: Group 1 (*N* = 10): 16 week old, Group 2 (*N* = 10): 22 week old, and Group 3 (*N* = 10): 25 week old with induced chronic inflammation (5 ng/kg LPS; 3X week). Each group was further segregated into pFUS stimulated (5 rodents with 2 wounds each) or sham control (5 rodents with 2 wounds each). One rat was removed from the Group 2 sham cohort during the study due to a hypertension complication. Rats were obtained from Charles River Laboratories (Wilmington, MA) and acclimated for 24 h. They were housed in 12 h:12 h light-dark cycle and were allowed *ad libitum* access to both food and water and were fed LabDiet 5008.

While under anesthesia using 2–3% inhaled isoflurane, two full thickness skin excision wounds were created on the head of each rat as follows. After shaving the head and cleaning the area with betadine and 70% ethanol, an 8-mm circular biopsy punch instrument (Acuderm, Fort Lauderdale, FL) was used to create the wounds, and the skin was carefully removed (Masson-Meyers et al., 2020). Analgesics were withheld due to the effects that available drug classes have on immune response (Huss et al., 2019); there were no outward signs of distress or discomfort observed throughout the course of the study. The wounds were left uncovered following surgery, and the rats were housed singly to avoid complications from the activity of cage mates. To induce chronic inflammation, rats in Group 3 received 5 ng/kg LPS 3X/week I.P. starting 14 days prior to wounding and continuing throughout the study.

Rats were anesthetized for all pFUS or sham treatments using 2–3% inhaled isoflurane, with each treatment procedure requiring approximately 10 min in total. Starting on day 1 post-surgery (24 h), pFUS energy was directed to the spleen after coupling a HIFU transducer to shaved skin with coupling gel. The spleen was identified by palpitation, and the probe was positioned toward the middle of the spleen. Wounds measured from anterior to posterior using digital calipers and the diameter was recorded. After the measurements were recorded, the wounds were photographed using a digital camera. To the groups receiving U/S stimulation, energy was applied for 3 1-min sessions with a rest of 30 s between each session as determined in a previous study (Cotero et al., 2019). The following ultrasound parameters were used: 1.1 MHz, 300 mV_*pp*_, 150 cycle burst, burst period of 200 ms (Cotero et al., 2019). In the case of sham controls, the transducer was identically placed over the spleen, but no energy was applied. Stimulations occurred on post-surgery days 1–4, 7–11, 14–15. Rats were euthanized on day 16 regardless of wound state. Figure 2 depicts the study timeline as outlined above.

Blood was drawn once a week and analyzed for both complete blood counts (CBC) (HemaVet; Drew Scientific) and blood chemistry (Preventative Care Profile Plus, VetScan; Abaxisis). Blood chemistry measurements, including glucose, were taken by 9:00 a.m. on sampling days. Remaining plasma was frozen for biochemical analysis. Upon completion of each study, rats were euthanized by CO_2_ inhalation. Following death, blood draws were completed *via* cardiac puncture and the spleens and wound beds were resected, snap frozen and stored at –80^°^C until cytokines were extracted and analyzed.

### Protein extraction for biochemical analysis

Frozen tissue was minced and added to ice-cold PBS supplemented with protease inhibitors (Roche, Little Falls NJ). Samples were homogenized using an IKA T18 Ultra-Turrax (IKA Works, Wilmington NC) set to 24,000 RPM until the tissue was dispersed. Samples were held on ice until all samples were processed and then tissue homogenates were cleared by centrifugation at 4^°^C. A NanoDrop (Thermo Fisher Scientific) instrument was used to assess the OD_280_ of each sample prior to each assay as outlined in the assay protocol.

### Cytokine screening

Panels of 34 cytokines, chemokines, and growth factors (gene symbols: AGER, AGRN, CCL2, CCL20, CD86, CNTF, CSF2, CX3CL1, CXCL1, CXCL2, CXCL3, CXCL5, FASLG, ICAM1, IFNG, IL10, IL13, IL1A, IL1B, IL1RL2, IL2, IL4, IL6, INHBA, LEP, MMP8, NGF, PDGFA, PPBP, PRLR, SELL, TIMP1. TNF, VEGFA) were assessed using a sandwich-based antibody array (C2 antibody array, RayBiotech, Norcross, GA). One hundred OD units of spleen or wound bed protein lysates were incubated with the membrane arrays and processed according to the manufacturer’s instructions with the exception that streptavidin-cy5 secondary antibody (Cytiva, Marlborough, MA) was used instead of streptavidin-HRP to utilize fluorescent scanning. Membranes were imaged using a Typhoon scanner (Cytiva, Marlborough, MA) and median fluorescence intensity values were extracted using ImageJ software (NIH) with the Microarray Profile (OptiNav Software, Bellevue, WA). All sample blocks were normalized to a reference block using positive control spots and background was subtracted using blank spots.

### Statistical analysis

We determined sample size using GraphPad StatMate version 2.00 (San Diego CA) and historical data for TNFα levels after LPS challenge (Cotero et al., 2019). With a standard deviation of 100. an *N* = 5 and a power of 80% the calculated difference of the mean that we would be able to detect is 291 pg/ml. our historical data demonstrated differences of over 500 pg/ml. Statistical analysis was performed using GraphPad Prism 9.4.1 (San Diego, CA). All results are expressed as means ± (standard error of the mean) SEM. Multiple unpaired Student’s *t*-test for healing rate and cytokine data or repeated measure two-way ANOVA followed by Tukey’s multiple comparisons test was used to assess the differences between groups for body weight, glucose levels, and wound size over time. Data were considered significant at *P* ≤ 0.05. Linear regression analysis of normalized wound diameter (percent of initial diameter) was used to extrapolate the time at which wounds would be completely closed. The x-intercept (days) when y = 0 (wound size) was defined to be the predicted time of closure. Cytokine arrays were compared by calculating the log2 fold change (Log2FC) of median fluorescence intensity for each pair of samples (pFUS vs. Sham). A heat map was generated and hierarchal cluster analysis was performed (Metsalu and Vilo, 2015) to demonstrate differences in groups. Pearson correlation comparing the relative expression of all 29 rats (regardless to group or treatment) and wound size was used to assess if there was a significant correlation between wound size and cytokine expression.

## Results

### Physiological characteristics

**FIGURE 3:**
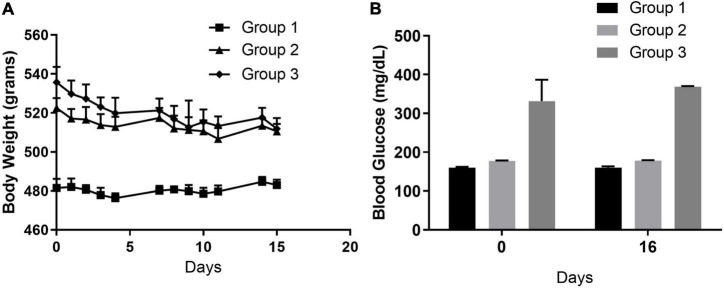
Physiological characteristics. **(A)** Body weight. Group 1 (16-week ZDSD prediabetic rats) no significant changes in body weight at 15 weeks. Similarly, Group 2 (22-week ZDSD prediabetic rats) did not have significant weight change. Group 3 (25 week diabetic + 10 ng/kg LPS) lost 4.6% body weight which was significant from day 0 (*p < 0.05) and likely due to the response to LPS. **(B)** Blood glucose levels. Random glucose levels were assessed on day 0 (prior to surgery) and day 16. No significant changes in were found in blood glucose levels in Group 1, 2, or 3 over the course of the study.

Physiological characteristics were assessed to track the overall health of the rats throughout the study. Rats were weighed each weekday day, and blood glucose was measured on day –14 (Group 3, when LPS administration started), day 0 (all groups) and day 16 (all groups). While body weights were not different between rats within the age-matched groups that received either pFUS or sham controls, they were different among the age groups (Figure 3A). Group 3, both pFUS and sham controls, exhibited significant weight loss, 5.7% (*P* < 0.05), during the study. This is to be expected due to low-grade chronic inflammation that LPS injections induced. Glucose levels were different for each group depending on age but remained unchanged between day 0 and day 16 (Figure 3B). Although CBC for each group were within normal range, significant differences in white blood cells (WBC) were observed in groups 1 and 2 both over time and between pFUS and sham treated rats (S1). Both monocytes and lymphocytes were lower in Group 1 at day 7, and neutrophils and monocytes were decreased by day 16 in Group 2. Significant differences were not observed in neutrophil, lymphocyte, or monocyte populations. Blood chemistry for each group were within the normal range in all measured parameters throughout the study (data not shown) with the exception of glucose as mentioned above.

### Wound size

**FIGURE 4:**
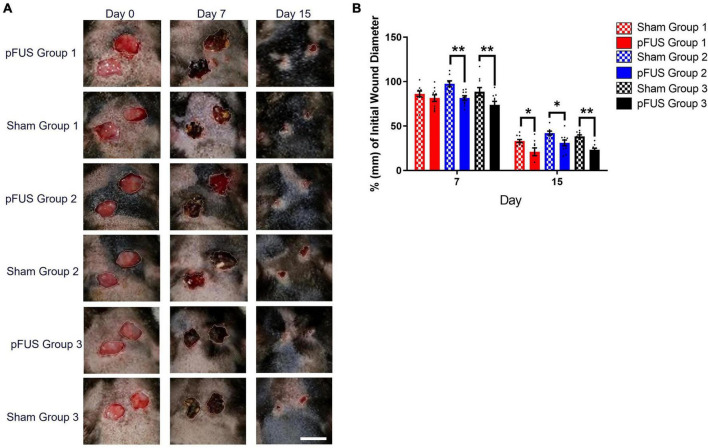
Wounds heal faster with Focused Ultrasound. **(A)** Representative longitudinal images of wound healing progression in ZDSD rats. Scale bar = 10 mm (lower right panel). **(B)** Wound diameters were normalized to their respective Day 0 diameter (E/B). Wounds were significantly smaller by day 7 in both Group 2 and Group 3 pFUS treated rats vs. Sham controls [Δ 15.94 ± 4.12% (*p* = 0.003) and Δ14.83 ± 6.02 (*p* = 0.024)]. At day 15, all pFUS treated were significantly smaller than sham (20 ± 4 vs. 33 ± 3; *p < 0.05) and week Group 3 treated were also significantly smaller than sham controls (24 ± 2 vs. 38 ± 2; **p < 0.001). *N* = 10 wounds/cohort.

Excisional wounds were created on day 0 (Figure 4A). Ultrasound stimulation or sham stimulation began approximately 24 h following the wounding procedure. Although an 8 mm biopsy punch was used to create defined circular wounds, there were nonetheless variations (≤ 1.5 mm; ≈14%) in wound diameter due to the looseness of the skin; consequently, all wound size measurements were normalized to the initial wound size. Group 1 pFUS treated rats demonstrated a reduction in wound diameter that was 4.68 ± 4.9% (ns) pFUS smaller than sham by day 7 and this difference increased to 12.16 ± 4.7% (*p* < 0.05) at day 15. Group 2 saw a decrease of 15.9 ± 4.1% (*p* < 0.05) (pFUS vs. sham) at day 7 and 10.97 ± 4.2% (*p* < 0.05) at day 15. Group 3 revealed a reduction of 14.83 ± 6% (*p* < 0.05) (pFUS vs. sham) at day 7 and 15.12 ± 2.2% (*p* < 0.001) at day 15 (Figure 4B). Sham controls (33–41% of original wound size at day 15) were comparable to the ZDSD from an earlier report by another group that observed ≈ 33% of original wound size at the same timepoint (Suckow et al., 2017).

**FIGURE 5:**
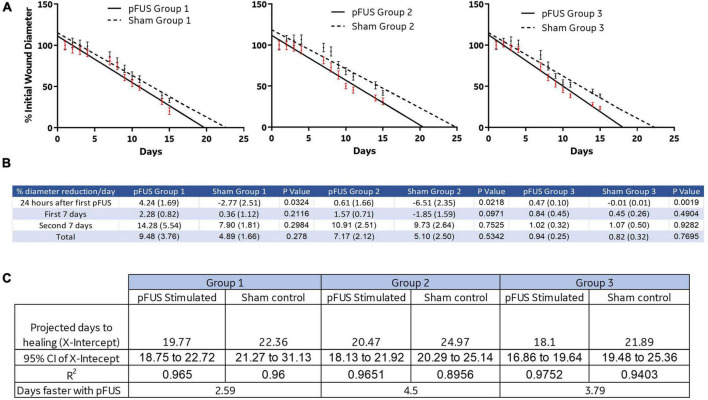
Linear regression and reduction of wound size. **(A)** Linear regression analysis was used to predict the days to healing which was reduced with pFUS stimulation in each group. **(B)** A rate of% reduction in diameter/day was calculated. The data suggest that pFUS influences the first 24 h of healing to a greater extent than the remainder of the time course. This may indicate that early wound bed inflammation is altered in a way that promotes healing. **(C)** Sham control animals in all disease progressions took between 22.36 and 24.97, while FUS stimulation resulted in healing times 2.6–4.5 days sooner.

While wounds closed in all groups over time, the rate of closure (% change in diameter/day), was significantly accelerated vs. corresponding sham controls in all three pFUS cohorts during the first 24 h after the first stimulation (Figure 5B). By 15 days, the closure rates were no longer different between pFUS and sham in any group As many wounds were not allowed to progress to complete closure, predicted time to healing (X-intercept) was determined by linear regression of the daily wound diameter measurements (Figure 5A). The elevation and intercepts of the slope were significantly different both within each group (Group1, *p* = 0.0032; Group 2, *p* = 0.0005; Group 3, *p* = 0.0012) and between groups (*p* < 0.001). At day 15, regression analysis suggested that wounds would be completely closed between days 17 and 23 (similar to healthy *SD* rats) for all pFUS stimulated cohorts as determined by the 95% CI of the X-intercept (days) when Y = 0 (wound size). Figure 5C demonstrates the regression analysis depicted in Figure 5A. The X-intercepts for the linear regression lines are designated as days to healing, and the days faster with pFUS are the difference between sham and pFUS at the X-intercept.

### Protein expression in spleen and wound bed lysates

**FIGURE 6:**
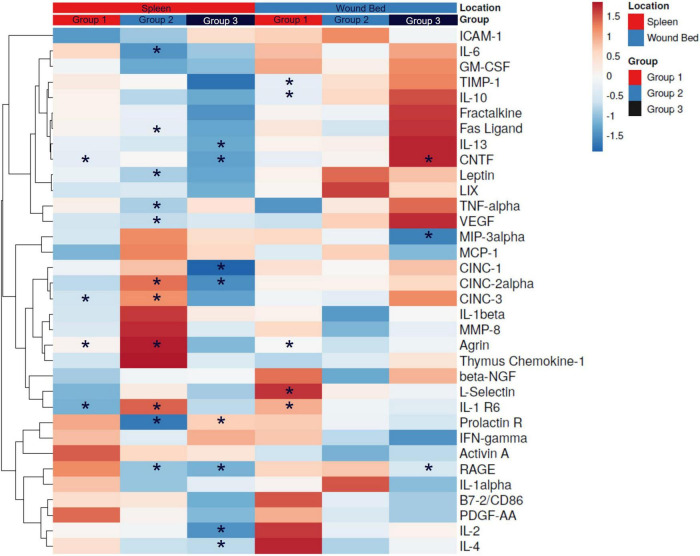
Cytokine expression in spleen and wound bed lysates. A. Spleen and wound bed lysates at day 16 post wounding were assessed against a panel of cytokines, chemokines and growth factors. A Heat map was generated depicting the relative change (log2 fold change of median florescence intensity) comparing pFUS vs. Sham controls. Hierarchical cluster analysis was performed where rows are centered; unit variance scaling is applied to rows. Rows are clustered using correlation distance and average linkage. Columns are grouped first by location followed by group. Asterisk represent significant differences in relative expression (*p < 0.05).

We examined the connection between splenic stimulation and distal wound sites by assaying both spleen and wound bed lysates against a panel of chemokines, cytokines, and growth factors. At 16 days following wounding, the relative changes in proteins were determined (log2FC) and plotted as a heatmap with hierarchal cluster analysis (Figure 6). Of the 34 proteins examined, spleen lysates from Group 1 had four proteins with significantly different expression levels [Agrin, 0.87 ± 0.24 (*p* = 0.004); CNTF, –0.58 ± 0.25 (*p* = 0.04) and IL-1R6, –1.20 ± 0.38 (*p* = 0.01)]; Group 2 had 11 proteins with significant differences from sham controls [Agrin, 3.10 ± 0.78 (*p* = 0.003); CINC-2α, 1.59 ± 0.67 (*p* = 0.046); CINC-3, 1.64 ± 0.68 (*p* = 0.04); Fas Ligand, –0.85 ± 0.28 (*p* = 0.01); IL-1 R6, 2.32 ± 1.03 (*p* = 0.047); IL-13, –0.71 ± 0.28 (*p* = 0.03); IL-6, –2.24 ± 0.81 (*p* = 0.02); Leptin, –1.22 ± 0.42 (*p* = 0.02); Prolactin R, –2.93 ± 1.24 (*p* = 0.046); RAGE, –2.70 ± 0.95 (*p* = 0.02); TNFα, –0.85 ± 0.31 (*p* = 0.02); VEGF, –0.60 ± 0.24 (*p* = 0.03)]; and Group 3 had 8 significantly altered protein levels [CINC-1, –1.98 ± 0.70 (*p* = 0.02); CINC-2α, –1.61 ± 0.71 (*p* = 0.049); IL-13, –1.29 ± 0.50 (*p* = 0.03); IL-2, –1.45 ± 0.47 (*p* = 0.01); IL-4, –1.53 ± 0.62 (*p* = 0.03); Prolactin R, 0.14 ± 0.06 (*p* = 0.04); RAGE, –2.95 ± 0.31 (*p* = 0.00001)]. Wound lysates from Group 1 had five proteins with different expression levels [Agrin, 0.68 ± 0.28 (*p* = 0.03); IL-1 R6, 1.64 ± 0.42 (*p* = 0.001); IL-10, –0.57 ± 0.24 (*p* = 0.03); L-Selectin, 2.53 ± 0.72 (*p* = 0.003); TIMP-1, –0.43 ± 0.18 (*p* = 0.03)]; Group 2 wound bed lysates were not found to have any significant differences and Group 3 had three proteins levels that were different [CNTF, 2.02 ± 0.79 (*p* = 0.03); MIP-3α, –1.93 ± 0.67 (*p* = 0.02); RAGE, –2.14 ± 0.90 (*p* = 0.04)]. Group 1 had two proteins that had significantly altered expression in both the spleen and wound bed (Agrin, increased in both and IL-1R6, down in spleen and up in wound) and Group 3 had one (RAGE, decreased in both spleen and wound bed). The dendrogram, which includes all 3 test groups and both spleen and wound bed lysate data, revealed 3 distinct clusters of closely associated proteins consisting of the following proteins in each: Cluster 1. ICAM-1, IL-6, GM-CSF, TIMP-1, IL-10, Fractalkine, Fas Ligand, IL-13, CNTF, Leptin, LIX, TNFα, and VEGF. Within this cluster the 2 proteins with the closest linkage were Fas Ligand and Fractalkine. Cluster 2. MIP-3α, MCP-1, CINC-1, CINC-2α, CINC-3, IL-1β, MMP-8, Agrin, Thymus Chemokine-1, β-NGF, L-Selectin, and IL-1R6. This cluster revealed the closest linkage between the CINC proteins. Cluster 3. Prolactin R, IFN-γ, Activin A, RAGE, IL-1α, B7-2/CD86, PDFG-AA, IL-2, and IL-4. In Cluster 3, the 2 closest proteins were B7-2/CD86 and PDGF-AA.

Table 1 summarizes both the proteins associated with, and their role in, wound healing along with which main cluster they were segregated into. Proteins measured in this study were associated with every stage and process of the wound healing cascade and many of them had significant alterations.

**TABLE 1:** Cytokines, chemokines and growth factors and their role in wound healing.

Phase	Process	Wound resolution involved proteins
Inflammatory	Inflammation	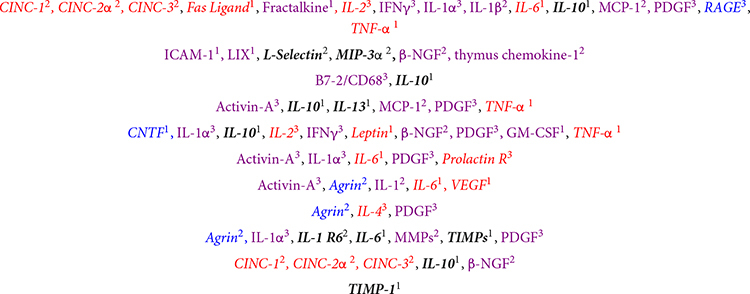
	Cell migration	
	Cell differentiation	
	Immune response	
Proliferative	Proliferation	
	Reepithelization	
	Angiogenesis	
	ECM tissue formation	
Remodeling	ECM remodeling	
	Wound contraction	
	Scaring	

The analytes tested in our panel listed with their role in wound healing. The proteins that were significantly different in pFUS vs. sham wound bed lysates at 16 days post wound creation are in italics. Analytes from each phase of healing were significantly altered as indicated by color (Group 1, red; Group 2, blue; and Group 3, black) and many processes within the phases were affected as well. Superscripts indicate the cluster that the proteins were associated with in the hierarchical cluster analysis.

**FIGURE 7:**
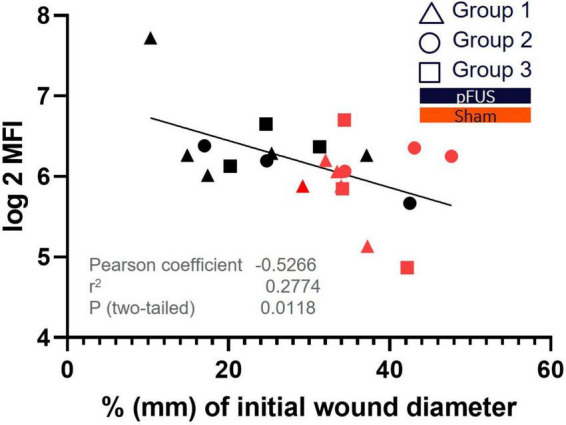
Correlation of Agrin expression to wound size. Agrin was identified as the only protein that had significant (*P* < 0.05) correlation with wound size. The scatterplot demonstrates a significant correlation between agrin protein expression in wound beds and% existing wound at day 15 post wounding. Higher agrin expression is exhibited in smaller (more closed) wounds which associates to its role in ECM formation and remodeling.

Another interesting observation was that agrin, which is associated with angiogenesis, ECM formation and ECM remodeling and more highly expressed in fibroblasts, monocytes and T-cells (Ambrožová et al., 2017), was the only protein from the panel that correlated, moderately, with wound diameter. A Pearson correlation matrix was used to examine all relative cytokine values with wound size (*n* = 29). Figure 7 demonstrates the negative correlation (Pearson coefficient –0.5266) between higher agrin protein expression with smaller wounds. Most of the wounds that were smaller were also in the pFUS stimulated groups (black markers) and had likely progressed toward proliferation and remodeling in the wound healing cascade.

## Discussion

The ZDSD rat model is a hybrid of the Zucker Diabetic Fatty (ZDF fa/fa) rat and *SD* (Peterson et al., 2015). The model recapitulates the stages of T2DM from pre-diabetes through overt diabetes to diabetic complications, and these rats have similar comorbidities as human T2DM including nephropathy, neuropathy, fatty liver, hypertension, metabolic syndrome, cardiac dysfunction, and chronic inflammation (Reinwald et al., 2009; Peterson et al., 2015, 2017). Non-fasting blood glucose levels are generally defined as < 140 mg/dL in cases of non-disease and > 200 mg/dL with diabetes, with the middle ground is classified as prediabetes (American Diabetes Association, 2010). Starting at week 21, non-fasting glucose levels spontaneously rise above 200 mg/dL in this rat model (Peterson et al., 2015). With the rise above euglycemic blood glucose levels in all 3 groups, we observed a characteristic delay in wound healing that was similar to published studies of the ZDSD model (Suckow et al., 2017).

Closure rates were significantly faster in pFUS treated rats during the 24 h following the first pFUS treatment (day 2 of the study). This corresponds to the time in the healthy healing cascade when the classical macrophage (M1) population is at its peak and neutrophils are post peak. We hypothesize that pFUS may facilitate the progression through the inflammation phase of healing *via* systemic cytokine level alteration. We have previously demonstrated, *via* measurements of splenic and whole blood levels of TNFα and splenic levels of IL-1α, that splenic pFUS modulates the systemic signaling of CAP and NF-KB in a rat model of endotoxemia (Cotero et al., 2019). We have also demonstrated that modulation can be connected between a locally stimulated lymph node and a the spleen *via* a long-range nerve pathway (Cotero et al., 2020), and that pFUS modulation of CAP attenuates the immune response in a mouse model of pneumonia (Ahmed et al., 2022). Lack of progression from a proinflammatory to an anti-inflammatory phenotype is one of the major deterrents of normal wound healing. The data presented in this report suggest that spleen-targeted pFUS stimulation accelerates wound closure in ZDSD rats, rendering the wound-healing response as similar to that reported for healthy rats. Our study also suggests, *via* altered healing rates, that the systemic immune response is altered with stimulation, driving the response from proinflammatory to anti-inflammatory (M1 to M2 phenotypes) more quickly than observed in sham controls. This may be explained by a continuous replenishment of wound macrophages from activated circulating monocytes (Olingy et al., 2017). Furthermore, the total blood volume of a 500 g rat (average starting weight in this study) is approximately 31 ml, the blood volume of the spleen is around 5% of the total volume, or 1.5 ml and the blood flow rate to the spleen is 0.63 ml/min (Davies and Morris, 1993). During the 3 min of ultrasound stimulation 1.9 ml of the blood volume has circulated through the spleen in addition to the resident volume, resulting in about 11% of the blood volume exposed to neurotransmitter and signaling molecules released in the spleen as a result of ultrasound energy, and could account for differences in immune cell activation and cytokine expression.

Differential cytokine expression that was observed in this study gives an indication as to how splenic stimulation with ultrasound could be having a systemic effect on peripheral organs (skin) *via* activation of CAP. Reservoir monocytes may be activated *via* acetylcholine binding to α7 receptors upon splenic pFUS (lowering TNFα and other pro-inflammatory cytokines). As the wound heals, and the spleen is stimulated daily, there may be an increased net infiltration of monocytes to the wound bed vs. non-stimulated sham controls. pFUS treatment may also allow these cells to be recruited more effectively from the splenic reservoir monocytes after they are released into circulation (Swirski et al., 2009). These mechanistic hypotheses will be examined in future studies, when earlier timepoints in the healing cascade are examined with the use of *in vivo* cell tracking to learn the fate, phenotypes, and timing of the immune cells after they are released from the spleen.

Cytokines, chemokines, and growth factors are central factors in orchestrating the wound healing response (Barrientos et al., 2008; MacLeod and Mansbridge, 2016; Ambrožová et al., 2017), and here we report several that are modulated by the splenic pFUS treatment. Specifically, high expression of RAGE is linked to inflammation, hyperglycemia, Alzheimer’s disease, cancer, and aging. When blocked or down regulated, inflammatory cell influx, NF-KB signaling, and cytokine production are suppressed (Ramasamy et al., 2009; Hudson and Lippman, 2018; Reed et al., 2020). RAGE, significantly lower in Groups 2 and 3 spleen and Group 3 wound beds in pFUS treated rats vs. corresponding sham rats, is involved with the inflammation phase of healing and is highly expressed in T and B lymphocytes as well as macrophage (Hindawi, 2021). L-selectin, expressed on leukocytes and responsible for mediating capturing and tethering to the vascular endothelium to traffic lymphocytes and neutrophils to inflammatory sites (Raffler et al., 2005), was significantly higher in the wound beds of Group 1. Ciliary neurotrophic factor (CNTF) has been shown to protect against LPS induced endotoxemia and reduce TNFα production (Benigni et al., 1995), and may also increase M2 macrophage differentiation (Blanco et al., 2019). The cytokines that were altered in the spleen after 2 weeks of ultrasound stimulation included IL-6, IL-13, CNTF, TNFα, CINC-1, CINC-2α, CINC-3, IL-1R6, and RAGE and are all expressed in the spleen as well as secreted into blood *via* leukocytes. Because these proteins are secreted, they are likely to effect systemic response to injury. IL-1R6 is expressed in predominately in skin but is also expressed in CD4^+^ T-cells and monocytes, but not neutrophils. IL-1R6 activation induces NF-kB and MAPK signaling, both which are essential for healing progression. Surprisingly, IL-10, a potent anti-inflammatory cytokine, was lower in both spleen and wound bed lysates in some groups. A possible reason is that by day 16 the wounds have progressed past the inflammatory phase, where IL-10 is most prevalent. Both agrin and IL-1 R6, involved with ECM remodeling were increased with pFUS vs. sham controls, while TIMP-1, implicated in scarring/fibrosis was decreased. Differences in expression of any particular cytokine, between groups, could be due to different wound healing progression as it relates to the severity of T2DM modeled with each group. Taken together, cytokines, chemokines and growth factors are responsible for many processes in the wound healing cascade and aberrant expression disrupts the progression through the healing cascade. pFUS stimulation of splenic CAP alters proteins associated with each phase of wound healing, as well as many processes within the phases. These alterations include lower expression of some proinflammatory proteins as well as increased expression of some anti-inflammatory proteins. To further elucidate the role of altered cytokines, future studies will examine the mechanisms by which events stimulated by pFUS in the spleen may be connected to alterations in wound healing at distant sites.

While this study was conducted in a rat model of T2DM, it is important to state that although we observed differences in would size, healing rates, and certain cytokine levels, the wounds created could not be classified as chronic wounds (wounds without healing progression for over 3 months). Treatment of the wounds started 24 h after wound creation while the wounds were still fresh, and it is very difficult to model chronic wounds in rodents.

## Data availability statement

The raw data supporting the conclusions of this article will be made available by the authors, without undue reservation.

## Ethics statement

This animal study was reviewed and approved by GE Research IACUC.

## Author contributions

CP was the principal investigator. JA was the co-PI of the study. CM and VC designed and conducted the study. FG contributed to drafting the original manuscript. All authors contributed to the article and approved the submitted version.
